# Development of Spherical Nucleic Acids for Prostate Cancer Immunotherapy

**DOI:** 10.3389/fimmu.2020.01333

**Published:** 2020-07-08

**Authors:** Lei Qin, Shuya Wang, Donye Dominguez, Alan Long, Siqi Chen, Jie Fan, Jihae Ahn, Kacper Skakuj, Ziyin Huang, Andrew Lee, Chad Mirkin, Bin Zhang

**Affiliations:** ^1^Division of Hematology/Oncology, Department of Medicine, Robert H. Lurie Comprehensive Cancer Center, Northwestern University Feinberg School of Medicine, Chicago, IL, United States; ^2^Interdisciplinary Biological Sciences Graduate Program, Northwestern University, Evanston, IL, United States; ^3^Department of Chemistry, Northwestern University, Evanston, IL, United States; ^4^Department of Materials Science and Engineering, Northwestern University, Evanston, IL, United States; ^5^Department of Chemical and Biological Engineering, Northwestern University, Evanston, IL, United States; ^6^The International Institute for Nanotechnology, Northwestern University, Evanston, IL, United States

**Keywords:** prostate cancer, vaccines, Immunostimulatory Spherical Nucleic Acids (IS-SNAs), CpG, immunotherapy

## Abstract

Although the strategy of therapeutic vaccination for the treatment of prostate cancer has advanced to and is available in the clinic (Sipuleucel-T), the efficacy of such therapy remains limited. Here, we develop Immunostimulatory Spherical Nucleic Acid (IS-SNA) nanostructures comprised of CpG oligonucleotides as adjuvant and prostate cancer peptide antigens, and evaluate their antitumor efficacy in syngeneic mouse models of prostate cancer. IS-SNAs with the specific structural feature of presenting both antigen and adjuvant CpG on the surface (hybridized model (HM) SNAs) induce stronger cytotoxic T lymphocyte (CTL) mediated antigen-specific killing of target cells than that for IS-SNAs with CpG on the surface and antigen encapsulated within the core (encapsulated model (EM) SNAs). Mechanistically, HM SNAs increase the co-delivery of CpG and antigen to dendritic cells over that for EM SNAs or admixtures of linear CpG and peptide, thereby improving cross-priming of antitumor CD8^+^ T cells. As a result, vaccination with HM SNAs leads to more effective antitumor immune responses in two prostate cancer models. These data demonstrate the importance of the structural positioning of peptide antigens together with adjuvants within IS-SNAs to the efficacy of IS-SNA-based cancer immunotherapy.

## Introduction

Prostate cancer is the second leading cause of cancer related death in the United States in men. In 2020, the estimated diagnosis of new cases is 191,930 (1). For patients with metastatic or recurrent prostate cancer, the benefits of chemotherapy, and androgen-deprivation therapy are limited (2–4). Therefore, new therapeutic approaches are of great interest for the large population of prostate cancer patients. The approach of cancer immunotherapy led to the generation of Sipuleucel-T, an FDA-approved cell-based vaccine for treating metastatic castration-resistant prostate cancer (mCRPC); Sipuleucel-T involves the treatment of patient antigen presenting cells (APCs) with a fusion protein of prostate-specific acid phosphatase (PAP) and GM-CSF, serving as adjuvant. In spite of benefits to overall survival, Sipuleucel-T did not result in benefits in progression-free survival (5). PROSTVAC, which uses a viral vector to induce the expression of PSA as antigen and three co-stimulatory molecules (6), is another example of an approach to therapeutic vaccination for prostate cancer. However, a phase 3 clinical trial using PROSTVAC as a monotherapy was suspended due to inadequate outcomes in overall survival (7). In addition to the unsatisfactory clinical outcomes of these vaccines, the production of these vaccines is complicated, time consuming, and costly.

Peptide-based therapeutic cancer vaccines, in contrast to cell-based therapies and vaccines based on viral vectors or plasmids, offer advantages in safety and scalable production (8). Clinical outcomes for peptide vaccines have been poor however. Among the major reasons for poor outcomes are the induction of T cell tolerance, caused by the low efficiency of co-delivering antigen and adjuvant molecules to dendritic cells (DCs) in draining lymph nodes (dLNs) (9), and T cell dysfunction and deletion due to the persistent antigen at vaccination sites (10).

Spherical nucleic acids (SNAs) are a class of nanostructures emerging as versatile therapeutic agents that can be used to treat a wide variety of diseases via gene regulation and immunotherapeutic pathways (11–16). They are attractive because they are novel structures with privileged access to tissues and cells that conventional linear nucleic acids cannot address without the use of ancillary transfection agents (17–21). Toll-like receptor (TLR) specific adjuvants, such as bacterial DNA or synthetic oligodeoxynucleotides (ODN) containing unmethylated CpG can provide more efficient and cell-specific activation signals to DCs which express the CpG receptor Toll-like receptor 9 (TLR-9), compared with standard adjuvants (e.g., incomplete Freund adjuvant) (22). CpG is a powerful TLR-9 agonist that activates DCs and promotes efficient antigen presentation, and subsequent priming of tumor antigen-specific CD8^+^ T cells (23). SNAs comprised of such sequences show greater potency or larger therapeutic windows than linear sequences at identical concentrations in cellular, animal, and human studies (14, 24, 25). Liposomal SNAs are a particularly promising class of constructs that present and outwardly orient synthetic nucleic acids at high surface density on a liposomal core (14, 26). As adjuvants, these structures are comparably potent to their prototypical gold based analogs (14), but are biocompatible and have advanced to several human clinical trials (14, 24). Moreover, the liposomal core provides a means for surface-presenting the CpG oligonucleotides and antigen with control over stoichiometric ratios, and liposomal SNAs are thus powerful vaccine candidates with highly tunable properties (14, 25). With the aim of generating effective antigen-specific CD8^+^ cytotoxic T lymphocyte (CTL) responses against prostate cancer, we designed two different SNA structures formulated with peptides derived from different prostate tumor-associated antigens and evaluated their efficacy as vaccines in tumor-free and prostate tumor settings, compared to those by an admix of linear CpG and peptide antigen.

In this study, we examined the ability of IS-SNAs to induce antitumor CD8^+^ T cell immune responses against three well-established prostate tumor-associated antigens (TAAs): prostate-specific antigen (PSA), prostate-specific membrane antigen (PSMA), and prostate acidic phosphatase (PAP). In our use of peptide antigen derived from PSA, we found that both EM and HM SNAs enhance uptake of CpG and antigen by DCs, when compared to that for the admix of CpG and peptide, and thereby promote the activation and cross-priming ability of DCs. We found that the position of the peptide within SNAs is a structural feature in vaccine design that influences the quality of antigen-specific immune responses. Notably, HM SNAs enhanced CTL mediated antigen-specific killing of target cells to a much greater extent than EM SNAs and admix groups. SNAs functionalized with prostate tumor-associated antigens (PSMA, or PAP) resulted in tumor inhibition upon immunization with HM SNAs in both RM1-PSMA and TRAMP-C2 (PAP) prostate cancer models. Our study therefore demonstrates the therapeutic potential of SNA structures varied in the position of peptide antigens within SNAs toward the development of SNA vaccines for prostate cancer immunotherapy. These findings are a critical advance in the development of IS-SNAs for cancer immunotherapy specifically for prostate cancer, a weakly immunogenic cancer, and are a major advance beyond previous studies showing immune responses limited to model antigens [e.g., ovalbumin (OVA)] (25).

## Methods

### Animals and Cell Lines

C57BL/6 mice, 6–8 weeks old, were purchased from Jackson Laboratory. Mice were used according to the protocols approved by institutional animal use committee at Northwestern University. Murine TRAMP-C1 prostate cancer cell line engineered to express human PSA (TRAMP-PSA) was kindly provided by Dr. Jeffrey Medin at University of Toronto (27), TRAMP-C2 prostate cancer cell line was generously provided by Dr. Barbara Foster at Roswell Park Institute, and human PSMA expressing RM1 prostate tumor cell line (RM1-PSMA) was generously provided by Dr. Michael Mathis at Louisiana State University (28). Both TRAMP cell lines were cultured in complete DMEM medium (10% fetal bovine serum, 100 U/mL penicillin G sodium and 100 μg/mL streptomycin) containing insulin from bovine pancreas (5 μg/ml, sigma) and dihydrotestosterone (10 nM, sigma) at 37°C in a 5% CO_2_ incubator. RM1-PSMA cell line were maintained in complete DMEM medium with G418 (200 μg/ml). B16F10 cells were cultured in complete RPMI-1640 medium supplemented with 5% fetal bovine serum.

### SNA Synthesis and Characterization

H-2D^b^-restricted peptide PSA_65−73_ (HCIRNKSVI), PSMA_634−642_ (SAVKNFTEI), PAP_115−123_ (SAMTNLAAL), and Gp100_25−33_ (KVPRNQDWL) were synthesized by GenScript (29, 30). 3′-cholesterol-functionalized CpG 1826 ODNs were synthesized using automated solid support phosphoramidite synthesis. Lipid (DOPC) was purchased from Avanti Polar Lipids. Liposome cores of SNAs were prepared as previously described (26). Briefly, pre-dried lipid films of DOPC were hydrated with solutions of phosphate buffered (PBS), or solutions containing peptides (1 or 2 mg/ml) for EM SNAs. The size of liposomes was controlled by freeze-thaw cycles and extrusion. Unencapsulated peptide was removed by dialysis or tangential flow filtration (100 kD membrane, Repligen). The final DOPC lipid and peptide concentrations of liposome core were determined by spectroscopic analysis using commercially available reagent kits for DOPC (Phosphatidylcholine assay kit, Sigma) or for peptides (Pierce quantitiative fluorometric peptide assay, ThermoFisher). Maximum values of the stoichiometry of peptide encapsulation for PSA_65−73_, PSMA_634−642_, and PAP_115−123_ were 75, 75, and ~15–20 per liposome core, respectively; lower levels of peptide encapsulation (~38 per liposome core) for PSA_65−73_ and PSMA_634−642_ were obtained by using lower concentrations of peptides during liposome formation. To conjugate peptides with oligonucleotides for HM SNAs, we used disulfide exchange for cysteine-containing peptides, and biochemically labile linkers previously reported by our group for non-cysteine peptides (16). Oligonucleotide-antigen conjugates were then purified by PAGE gel in 1X TBE buffer. Functionalization of liposomes with CpG (for EM SNAs) or CpG hybridized to oligonucleotide-antigen conjugates (for HM SNAs) was accomplished by the adsorption of 3′-cholesterol-CpG ODNs with liposomes at a ratio of ~75 CpG oligonucleotides per liposome. Concentrations of CpG used in these adsorption reactions were measured by absorbance spectroscopy. The concentration of CpG formulated into purified SNAs was confirmed by absorbance spectroscopy and analysis by agarose gel electrophoresis that showed the absence of oligonucleotides that were not associated with liposomes (25). SNAs with PAP_115−123_ were functionalized with a mixture of CpG and non-stimulatory (dT)_20_ oligonucleotides in order to generate SNAs with the desired PAP_115−123_:CpG ratio while keeping the total oligonucleotides per liposome to be ~75 (for both EM and HM SNAs).

The size of SNAs and extruded liposomes was measured by DLS as described previously (26). Briefly, SNAs and liposome cores were diluted in PBS at ~10–100 nM in liposome concentration. The average size was calculated based on five measurements at 25°C. The polydispersity index (PDI) was calculated as the width of the size distribution using cumulants analysis. For Cryo-EM analysis, SNA samples were cast onto copper grids with lacey carbon using FEI Vitrobot Mark III. The grid was imaged using a Hitachi HT7700 TEM with a Gatan cryo-transfer holder. To analyze the loss of peptides from EM SNAs, FITC labeled peptides were encapsulated into SNAs. Upon incubating SNAs in media containing FBS (10%) at 37°C (shaking at 300 rpm), we examined the release of encapsulated peptides by measuring the fluorescence of solutions obtained by filtering SNA samples through a 30-kDa cutoff filter. Quantification of released peptide was determined by fluorescence with the fluorescence of standards prepared from the peptide solutions.

### BMDC Uptake and Activation

Bone marrow derived dendritic cells (BMDCs) were generated by culturing mouse femurs bone marrow cells in complete RPMI-1640 medium containing GM-CSF (20 ng/ml, Biolegend). The medium was half replaced every 2 days. On day 6, the loosely attached DCs were collected and phenotyped before experiments. For BMDC uptake, cells were treated with Cy5-labeled CpG and TMR-labeled PSA_65−73_ in EM, HM, or admix formulations for 1 h. The uptake was evaluated using confocal microscope (Zeiss LSM 800) or flow cytometer (BD LSR II). To analyze the colocalization of fluorescent labeled CpG and peptide inside the DCs, 8 to 10 single images were randomly picked. The colocalization was quantified using Manders coefficient (10 Z-stack images of each cell). To evaluate the activation status of BMDCs, cells were incubated with different formulations for 0.5 h, and the activation markers were measured 8 h after initiating incubation by flow cytometry. The flow cytometry data was analyzed by FlowJo software, and the gate was set on CD11c^+^ cells.

### *In vivo* DC Uptake, Activation, and Cross-Presentation

Naïve C57BL/6 mice were injected subcutaneously with different formulations containing PSA_65−73_ (3 nmol) and CpG (6 nmol) with or without fluorescence labeling. For uptake study, mice were sacrificed and inguinal lymph nodes were harvested 2 h post injection. The LNs were processed to single cell suspension by passing it through a 70-μm cell strainer (Fisher Scientific) for flow cytometry analysis.

For DC activation and cross-presentation study, LNs were harvested 24 h after injection and single cell suspension was prepared. The lymphocytes were counted. Part of the lymphocytes were stained with fluorophore-labeled antibodies against CD11c, CD11b, CD8, CD40, CD80, and CD86 for activation status analysis. To evaluate the cross-presenting capacity of DCs, the same amount of lymphocytes from each mice of the same group was combined and DCs were purified using biotin anti-mouse CD11c antibody in conjunction with EasySep mouse biotin selection kit (Stemcell Technologies). The PSA_65−73_ antigen-specific T cells were generated by prime-boost immunization of naïve mice with EM SNA/PSA_65−73._ Purified DCs were then co-cultured with purified PSA_65−73_ specific CD8^+^ T cells (10^5^/well) at 1:2 ratio for 48 h in 96-well-plate. The antigen-specific CD8^+^ T cells alone were used as controls. The cross-presenting capacity of DCs was determined by number of IFN-γ spots using ELISPOT assay (mouse IFN-γ ELISPOT Ready-SET-Go kit, eBioscience). For cytokine production, the supernatant from DC and T cell co-culture was evaluated by luminex assay (R&D Systems).

### Immunization and Cancer Immunotherapy Experiment

To evaluate the antigen-specific T cell response generated by SNAs, naïve C57BL/6 mice were subcutaneously immunized with EM SNAs, HM SNAs, or admix of peptide and CpG (3 nmol PSA_65−73_/6 nmol CpG, or 6 nmol PSMA_634−642_/6 nmol CpG per mouse) in 100 μl volume every 2 weeks for 3 times. One week after final immunization, mice were sacrificed and spleens were harvested for T cell assessment. IFN-γ ELISPOT assay was performed as described previously using splenocytes from immunized mice (31). CD8^+^ T cell restimulation and intracellular staining for IFN-γ staining were carried out as published previously with minor modification (32). Briefly, splenocytes were cultured at 37°C for 4 h in the presence of peptide (10 μg/ml), monensin (2 μM), BFA (10 μg/ml), PMA (50 ng/ml), Ionomycin (1 μg/ml), and CD107a antibody (0.5 μg). For T cell phenotypic analysis, splenocytes were stained using antibodies against CD4, CD8, CD44, and CD62L. The cytotoxicity of CD8^+^ T cells were tested using *in vitro* CTL cytotoxicity assay.

For therapeutic cancer vaccine studies, 3 × 10^6^ TRAMP-C2 or 10^5^ RM1-PSMA prostate cancer cells were injected subcutaneously to the male mice on the right flank. Mice were then immunized subcutaneously with different formulations of peptide and CpG (6 nmol PAP_115−123_/6 nmol CpG, or 6 nmol PSMA_634−642_/12 nmol CpG per mouse) every week. The tumor growth was monitored every 2–4 days, and tumor volume was calculated as 0.5 × length × width × height. The fold change of tumors was calculated using the following formula: (tumor volume of last time point)/(tumor volume of 1st time point). In TRAMP-C2 tumor model, mice were sacrificed 41 days after tumor challenge, and tumors and spleens were harvested. Tumor tissues were weighed and digested in RPMI-1640 containing 1 mg/ml collagenase D (Roche) and 0.08 mg/ml DNase I (Roche) for 30 min at 37°C. Cells were then stained with anti-mouse CD45, CD8, and CD4 antibodies to analyze tumor infiltrating lymphocytes. Splenocytes were stained with antibodies against CD4, CD8, CD44, and CD62L for T cell phenotypic analysis.

### *In vitro* CTL Cytotoxicity Assay

The antigen-specific CTLs were first restimulated as described previously (33). Briefly, splenocytes from naïve syngeneic mice were γ-irradiated (3000 rad) and pulsed with peptide (10 μg/ml) to use as feeder cells. These cells were then incubated with splenocytes from immunized mice for 5 days at 1:4 ratio to expand antigen-specific CTLs. Half of culture medium was replaced with RPMI-1640 containing 20 ng/ml rhIL-2 (Biolegend) 2 or 3 days after co-culture. On day 5, cells were harvested and CD8^+^ T cells were selected (Stemcell Technologies). The CTL cytotoxicity was evaluated by killing of target cells based on the apoptosis status of target cells (34). Purified CD8^+^ T cells were co-cultured with efluor450 (eBioscience) labeled target cells (5 × 10^3^) at different effector to target (E/T) ratio in 96-well U-bottomed plate. The wells containing target cells only were used as controls. After overnight co-culture at 37°C, the cells were harvested and stained with 7-AAD (BD Bioscience) and Annexin V (Biolegend) for 15 min in the dark. CTL cytotoxicity (%) was calculated as cells positive for both eFluor450 and Annexin V/total eFluor450 positive cells, after subtracting the spontaneous apoptosis (%) in target cell only controls.

### Statistical Analysis

A two-tailed unpaired Student's *t*-test was used to compare difference between two groups. For tumor growth analysis among groups, two-way ANOVA with Bonferroni multiple comparisons post-test was used. A *p* < 0.05 was considered statistically significant. Data are presented as mean ± SEM in all experiments.

## Results

### Design, Synthesis, and Characterization of SNAs

**Figure 1:**
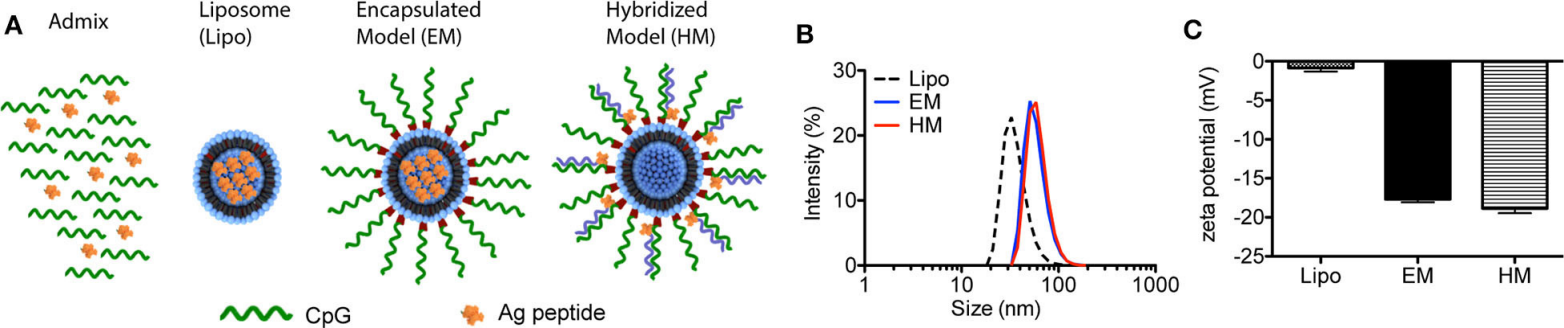
Characterization of IS-SNAs. **(A)** Diagram of Admix, liposomes, EM and HM IS-SNAs. **(B)** Dynamic light scattering analysis and **(C)** zeta potential of liposomes, EM IS-SNAs, and HM IS-SNAs.

The two different structures of liposome-based IS-SNAs (HM, EM) were prepared by functionalizing ~42 nm DOPC liposomes (formed by hydrating DOPC in aqueous buffer and extrusion through polycarbonate membranes) through the adsorption of CpG oligonucleotides modified with 3′-cholesterol groups (14, 26). The three H-2D^b^-restricted prostate TAA peptides used in our study are: human PSA_65−73_ (HCIRNKSVI), human PSMA_634−642_ (SAVKNFTEI), and mouse PAP_115−123_ (SAMTNLAAL); these peptides are derived from the antigens associated with prostate cancer, and have high binding affinity to murine MHC class I molecules. The peptides were incorporated into SNAs in two different ways. For EM SNAs, the peptide antigens are encapsulated within the liposomal core (prepared by hydrating DOPC in buffer solutions containing peptide, followed by extrusion) (Figure 1A). For HM SNAs, peptide antigens are chemically conjugated to oligonucleotides with a sequence complementary to CpG oligonucleotides and hybridized to CpG oligonucleotides with 3′-cholesterol groups; the peptide-functionalized double-stranded DNA is adsorbed to the surfaces of DOPC liposomes (16, 25). Both EM and HM SNAs showed spherical morphology with an average diameter of 52.5 ± 3.0 nm and 54.7 ± 2.5 nm, respectively, while liposomes exhibited an average diameter of 42.3 ± 2.9 nm (Figure 1B, Supplementary Figure 1A). Functionalization of liposomes with CpG led to structures with negative electrostatic charge as measured by the zeta potential, and thus the physical properties of EM and HM SNAs were similar (Figure 1C). The loss of encapsulated peptide from liposome cores was negligible, determined by our analysis of EM SNAs incubated in medium containing 10% FBS for 12 h (Supplementary Figure 1B).

### Enhanced Co-delivery of CpG and Antigen to Dendritic Cells by SNAs

**Figure 2:**
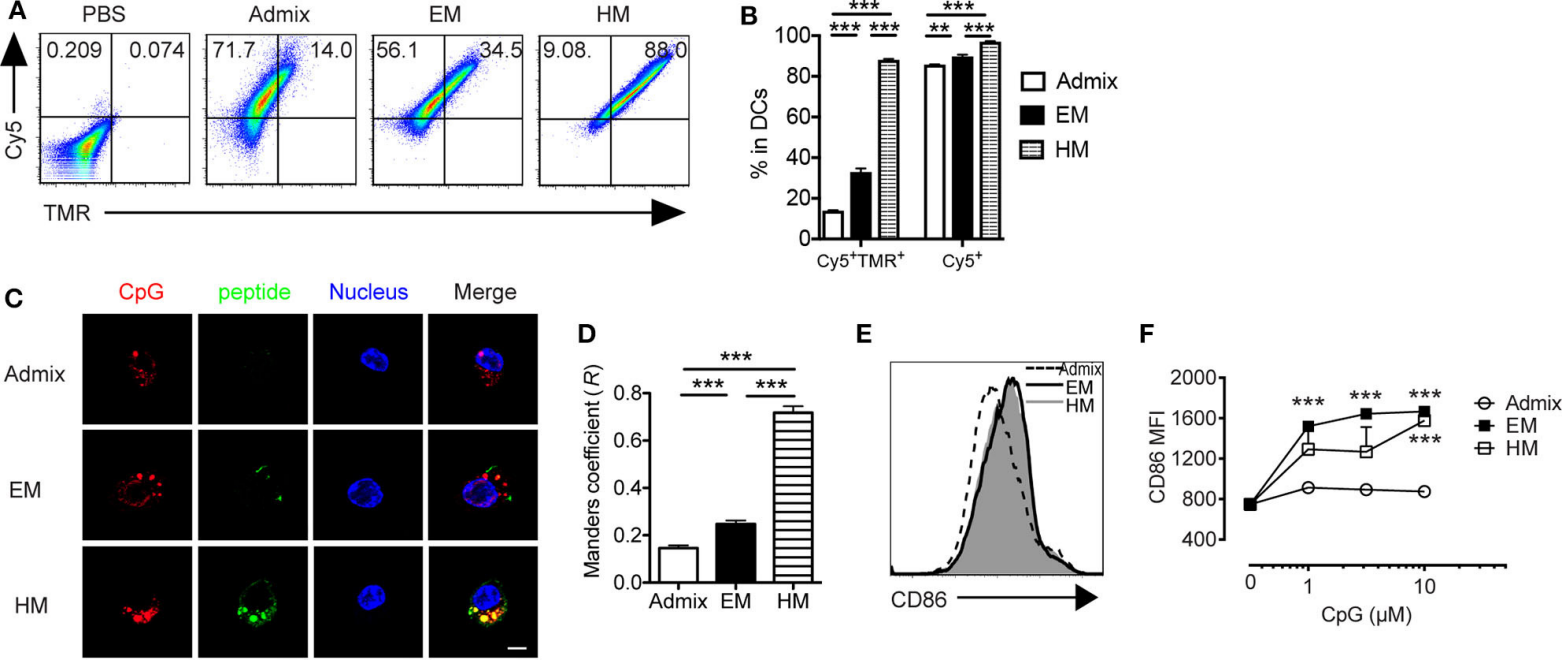
IS-SNAs facilitate DC uptake and activation *in vitro*. BMDCs were incubated with 5-TAMRA (TMR) labeled PSA_65−73_ and Cy5 labeled CpG in the form of admix, EM SNA, or HM IS-SNAs for 1 h. Uptake of IS-SNAs by BMDCs was monitored using flow cytometry **(A,B)** or confocal microscopy **(C,D)**, respectively. **(A)** Representative flow dot plots and **(B)** percentages of Cy5^+^TMR^+^, Cy5^+^ BMDCs receiving different formulations containing 50 nM CpG. **(C)** Confocal microscopy images of BMDCs. Scale bar, 5 μm. **(D)** Subcellular colocalization analysis of PSA_65−73_ and CpG in **(C)** using Manders coefficient (0.6 < *R* ≤ 1.0 indicates colocalization). **(E,F)** BMDC activation status was examined by expression of co-stimulatory molecule CD86 8 h after incubation. **(E)** Representative histograms for CD86 expression by BMDCs at 10 μM (by CpG). **(F)** Median fluorescent intensity (MFI) for CD86 by BMDCs treated with admix or IS-SNAs. Data are presented as mean ± SEM. **p < 0.01 and ***p < 0.001 analyzed by two-tailed unpaired Student's *t*-test.

Dendritic cells (DCs) are professional APCs that play a vital role in generating CTL responses. Their uptake of adjuvant and peptide, co-stimulatory molecule expression status (CD40, CD80, and CD86) and cytokine production, mainly IL-12, are key parameters that influence the magnitude of antitumor CTL responses (35). We evaluated the uptake of IS-SNAs formulated with fluorophore-labeled CpG and PSA_65−73_ by BMDCs, and the co-delivery of these components *in vitro*, and compared it to that for an admixture of linear CpG and free peptide. Upon 1 h of incubation of BMDCs with EM SNAs, HM SNAs, or admixture of Cy5-labeled CpG and TMR-labeled PSA_65−73_, we measured the uptake of CpG and peptide by BMDCs by flow cytometry. More than 80% of the BMDCs were Cy5^+^ upon treatment at 50 nM by CpG by flow cytometry (Figures 2A,B). The fraction of cells containing high levels of TMR-PSA_65−73_ was highly dependent upon the formulations of peptide and CpG used to treat the BMDCs; we observed increasing levels of TMR-PSA_65−73_ uptake in the order of admix < EM SNAs < HM SNAs. A key consequence of these treatments was the high percentage of CpG and peptide double positive (Cy5^+^ TMR^+^) BMDCs upon treatment with EM (33%) and HM SNAs (88%) (Figures 2A,B); these percentages were 1.4 and 5.3-fold higher than that for BMDCs treated with the admix control, respectively (Figures 2A,B, *p* < 0.001, *p* < 0.001). Analysis of the treated BMDCs by confocal fluorescence microscopy further confirmed the uptake of CpG and peptide. Both EM SNA- and HM SNA-treated BMDCs showed higher fluorescence intensity of TMR-PSA_65−73_ than BMDCs treated with the admix control, and HM SNA-treated cells showed higher levels of uptake of peptide than cells treated with EM SNAs (Figure 2C, Supplementary Figure 2B). Furthermore, the Manders coefficient value indicated high colocalization of CpG and peptide in HM treated BMDCs (>0.6), but not in admix or EM treated BMDCs (Figure 2D). These observations suggest that the success in the co-delivery of CpG and peptide by HM SNAs might be due to the entry of HM SNAs as intact structures; treatment with EM SNAs lead to comparably high levels of CpG uptake but lower levels of antigen delivery and co-localization, suggesting the release of TMR-PSA_65−73_ from EM SNAs. The enhanced co-delivery of CpG and peptide by SNAs led to significant up-regulation of the co-stimulatory molecules CD86 and CD40 compared to treatment with admixture of CpG and peptide (Figures 2E,F, Supplementary Figure 2C). Differences in CD86 and CD40 expression levels between EM and HM SNA treated groups were not significant.

**Figure 3:**
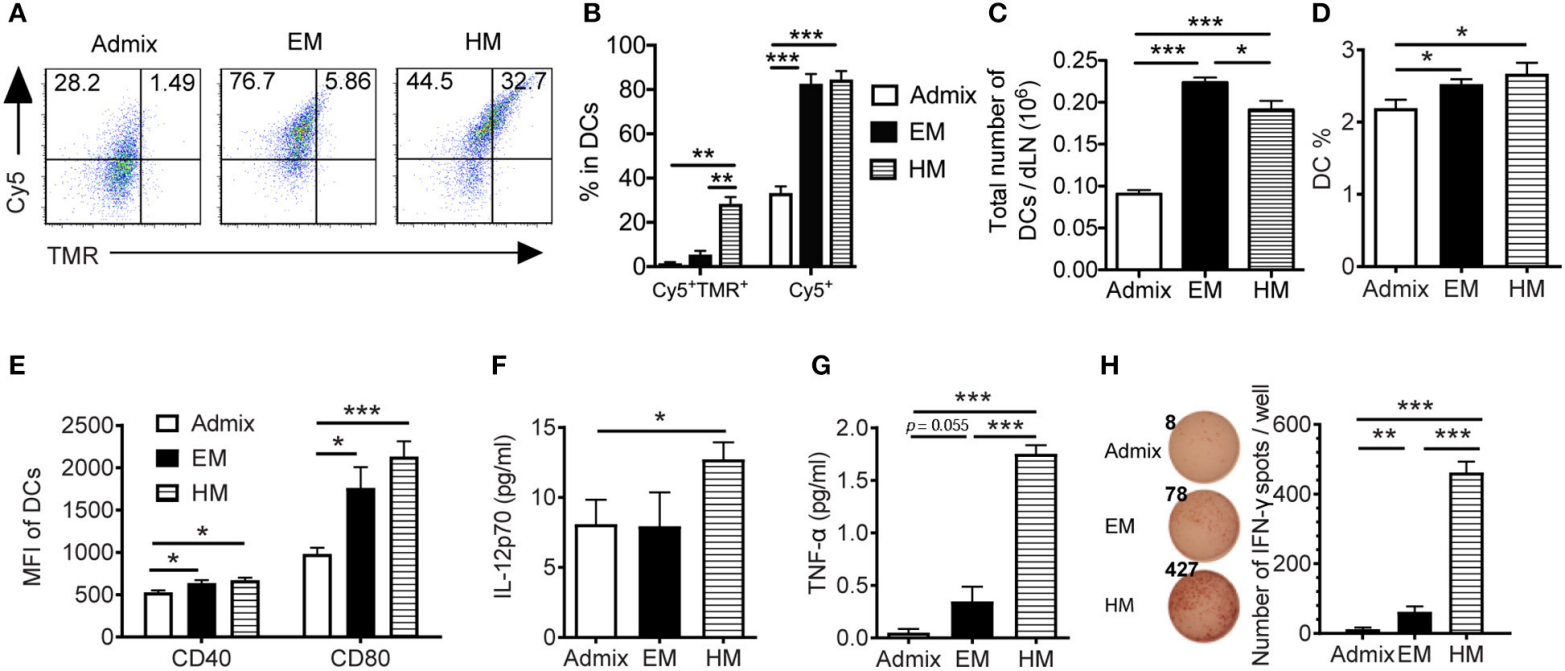
IS-SNAs enhances antigen and CpG co-delivery, thereby promoting DC activation and cross-presentation *in vivo*. **(A,B)** For co-delivery evaluation, mice were injected subcutaneously with TMR-labeled PSA_65−73_ and Cy5-labeled CpG in the form of admix, EM or HM IS-SNAs. Fluorescence signals of CD11c^+^ DCs in dLNs were measured after 2 h by flow cytometry. The uptake (3 mice per group) was shown by representative dot plots **(A)** and bar graph **(B)**. **(C–H)** For DC activation and cross-presentation, dLNs (4 mice per group) were harvested and analyzed 24 h after IS-SNA/PSA_65−73_ administration. Total number **(C)** and percentage **(D)** of CD11c^+^ DCs in dLNs and the expression of co-stimulatory molecule CD40 and CD80 **(E)** of these DCs were analyzed by flow cytometry. **(F–H)** Purified DCs from dLNs were co-cultured with PSA_65−73_ specific CD8^+^ T cells at 1:2 ratio for 48 h. The supernatant from the co-culture were harvested for analyzing IL-12 **(F)** and TNF-α **(G)** production. **(H)** The DC cross-presentation ability was evaluated using IFN-γ ELISPOT assay. All data are presented as mean ± SEM. *p < 0.05, **p < 0.01, and ***p < 0.001, analyzed by two-tailed unpaired Student's *t*-test.

To elicit a robust CTL response *in vivo*, SNA vaccines need to deliver peptide and CpG to the lymph nodes, where they are taken up by DCs and CTL responses are initiated (36). We examined the uptake of peptide and CpG formulated as SNAs by DCs in dLNs, and the function of these DCs *ex vivo*. Upon subcutaneous administration of SNAs formulated with TMR-PSA_65−73_ and Cy5-CpG for a 2 h period, DCs from the dLNs of both EM SNA- and HM SNA-treated mice showed 1.5-fold higher percentage of Cy5^+^ cells when compared with admix group, suggesting a greater uptake of CpG when using SNA formulations (Figures 3A,B, *p* < 0.001). The differences were more dramatic than those seen *in vitro*, where the vaccine materials were exposed to BMDCs in isolation (Figures 2A,B). DCs from HM SNA-treated mice showed the highest percentage of Cy5^+^TMR^+^ double positive DCs (17 and 4-fold higher than that of the admix and EM SNA treated groups), indicating a high yield of co-delivery of peptide and CpG to DCs (Figure 3B, *p* < 0.01). For optimal priming of antigen-specific T cells, DCs must present antigen peptide while displaying costimulatory molecules and producing pro-inflammatory cytokines (37). We found that the advantages in the co-delivery of CpG and antigen by HM and EM SNAs correlated with increasing numbers and fraction of CD11c^+^ DCs, over those observed for mice treated with the admix control (24 h following treatment, Figures 3C,D). We also found significant increases in the expression levels of co-stimulatory molecules CD40 and CD80 with SNA treatment (Figure 3E). In addition, we observed a significant increase in the percentage of CD11b^+^ DCs and CD8^+^ DCs, and their expression level of CD80 in EM and HM SNA-treated groups, compared to those of the admix group; differences between EM and HM SNA-treated groups were not significant (Supplementary Figures 3A,B).

To evaluate the ability of DCs from these immunized mice to cross-prime T cells, we co-cultured the CD11c^+^ DCs purified from dLNs with PSA_65−73_ antigen-specific T cells. We measured the secreted cytokines from co-culture supernatants using Luminex assays, and found significantly higher levels of secreted IL-p70 and TNF-α in wells containing DCs from HM SNA-treated mice than those from the admix group (Figures 3F,G, Supplementary Figure 3C). The co-cultures of T cells with DCs activated by HM SNAs *in vivo* produced more TNF-α and IL-12 than those from treatment with the CpG and peptide admix, and these cytokines play important role in priming effective CTL responses (38). DCs from EM and HM SNA-treated mice dramatically induced antigen-specific effector CD8^+^ T cell responses, with 4 and 38-fold greater numbers of IFN-γ expressing cells in the EM and HM SNA groups than the admix control group (Figure 3H); as controls, PSA_65−73_ -specific T cells without co-culturing with DCs did not produce IFN-γ (data not shown). These results indicate the importance of structure and the position of antigen within SNAs for raising DC-mediated antitumor T cell responses.

### Antigen-Specific T Cell Responses Generated *in vivo* by SNAs

**Figure 4:**
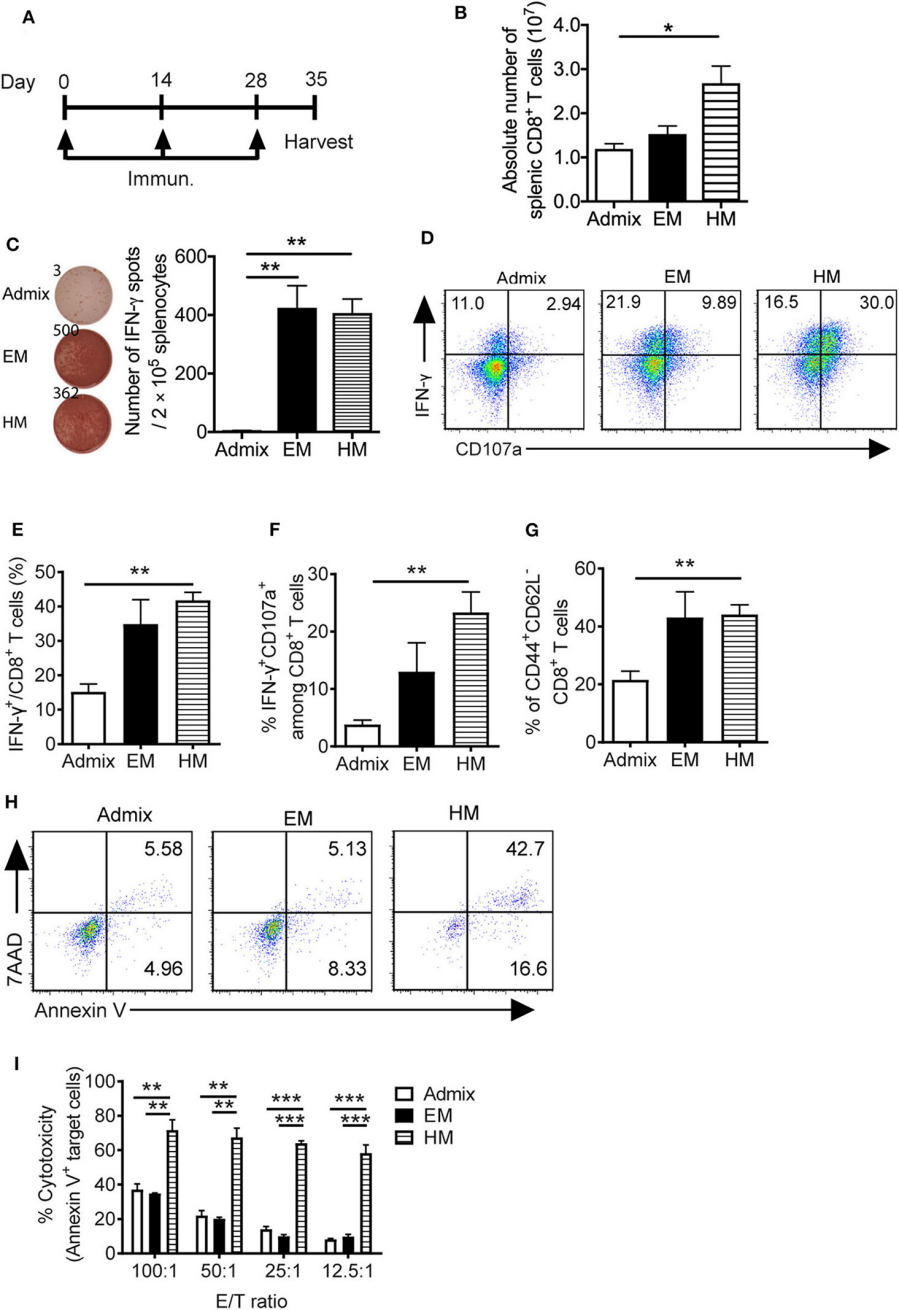
IS-SNAs promote PSA antigen-specific CD8^+^ T cell responses. **(A)** Immunization schedule of IS-SNA/PSA_65−73_. Mice (3 mice per group) were subcutaneously immunized with EM SNA, HM SNA, or admix formulation every 2 weeks for 3 times. Spleens were harvested for analysis 1 week after final immunization. **(B)** Absolute number of splenic CD8^+^ T cells from mice immunized 3 times with admix or IS-SNA/PSA_65−73_. **(C)** ELISPOT assay was used to measure PSA_65−73_ specific IFN-γ secreting cells by stimulating splenocytes with PSA_65−73_ peptide for 48 h. **(D–G)** Flow cytometry analysis of splenic CD8^+^ T cells. **(D)** Representative flow dot plots and bar graph of **(E)** IFN-γ and **(F)** IFN-γ and CD107a double positive CD8^+^ T cells. **(G)** Percentage of CD44^+^CD62L^−^ CD8^+^ T cells. **(H,I)** Splenic CD8^+^ T cell cytotoxicity to target cell TRAMP-PSA at different effector/target (E/T) ratio. **(H)** Representative flow dot plots at 12.5:1 E/T ratio. **(I)** Summary of CD8^+^ T cell cytotoxicity reflected by the percentage of apoptotic target cells. All data are presented as mean ± SEM. *p < 0.05, **p < 0.01, and ***p < 0.001, analyzed by two-tailed unpaired Student's *t*-test.

We evaluated the ability of SNAs to generate PSA_65−73_ specific T cell responses *in vivo*. We immunized mice with PSA_65−73_ and CpG in EM SNA, HM SNA, or admix formulations by subcutaneous injections every 2 weeks, for a total of 3 immunizations (Figure 4A). The antigen-specific T cell responses were characterized 7 days after the third immunization. Immunization with HM SNAs dramatically expanded the systemic T cells in the spleen compared with other treatment groups (Figure 4B). Splenocytes from EM or HM SNA-treated mice (but not admix-treated mice), when restimulated with PSA_65−73_, showed robust IFN-γ production on a per cell basis (*p* < 0.01, Figure 4C). This response was antigen-specific, as splenocytes stimulated with irrelevant peptide gp100 did not show any IFN-γ production (Supplementary Figure 4A). The activity of the CD8^+^ T cells from the three treatment groups showed a clear dependence on vaccine structure. Immunization with HM SNAs significantly increased IFN-γ producing CD8^+^ T cells compared with admix control (*p* < 0.01, Figures 4D,E). Vaccination with HM SNAs generated a larger percentage of poly functional T cells, observed by comparing the number of double positive cytokine producing (IFN-γ^+^) and degranulating (CD107a^+^) CD8^+^ T cells, with HM SNA/PSA generating the highest number (*p* < 0.01, Figures 4D,F); these cells are critical for controlling tumor growth. Furthermore, immunization with HM SNAs led to an increase in CD44^+^CD62L^−^ effector memory T cells when compared with admix group (*p* < 0.01, Figure 4G). In contrast, immunization with the admix of CpG and PSA_65−73_ induced a minimal antigen-specific CTL response in immunized mice.

We determined the killing capacity and specificity of the CD8^+^ T cells raised by SNA immunization for target cells expressing PSA. PSA-specific CD8^+^ T cells from immunized mice were expanded *in vitro* by co-culturing with PSA_65−73_ peptide pulsed APCs for 5 days. These CD8^+^ T cells were purified and co-cultured with PSA-expressing tumor cells (TRAMP-PSA) at ratios from 100:1 to 12.5:1. CD8^+^ T cells obtained from vaccination with HM SNAs were more cytotoxic against TRAMP-PSA cells than those from vaccination with EM SNAs or admixture of CpG and PSA_65−73_ (Figures 4H,I). At different effector/target (E/T) ratios tested (100:1, 50:1, 25:1, 12.5:1), CD8^+^ T cells from the HM SNA group displayed superior ability to kill target cells, compared with those from the EM SNA and admix groups. This killing is specific for PSA, as co-culturing of these CD8^+^ T cells with B16F10 tumor cells, which do not express PSA, led to negligible killing in all three groups (Supplementary Figure 4B). These observations show that immunization with HM SNAs leads to CD8^+^ T cells with greater killing capacity on a per cell basis than those from immunization with EM SNAs or admixtures of CpG and PSA_65−73_. Taken together with the greater number of CD8^+^ T cell in spleens from HM SNA groups (Figure 4B), these results suggest that immunization with HM SNAs would lead to superior anti-tumor activity *in vivo*.

We also examined the ability of EM and HM SNAs formulated with a peptide antigen for prostate-specific membrane antigen peptide (PSMA_634−642_) to trigger antigen-specific CTL responses *in vivo*. Similar to the results using PSA-conjugated SNAs above, 7 days after the third immunization, CD8^+^ T cells from both PSMA-conjugated EM and HM SNA immunized mice showed greater percentages of IFN-γ^+^ and IFN-γ^+^CD107a^+^ CD8^+^ T cells (*p* < 0.01, *p* < 0.001, Supplementary Figure 5A), along with significantly higher PSMA specific cytotoxicity against PSMA expressing target cells (RM1-PSMA) compared with admix control (Supplementary Figure 5C). Immunization with EM and HM SNAs also resulted in a significant increase in CD44^+^CD62L^−^ effector memory T cells when compared with admix group (*p* < 0.01, *p* < 0.001, Supplementary Figure 5B). These results show that immunization with HM SNAs induces superior antigen-specific antitumor CD8^+^ T cell responses for both two prostate tumor-associated antigens examined, and show that the effectiveness of SNA structures in inducing CTL responses is not limited to model antigens (e.g., OVA, E6) (25).

### Prostate Tumor Inhibition by SNAs

**Figure 5:**
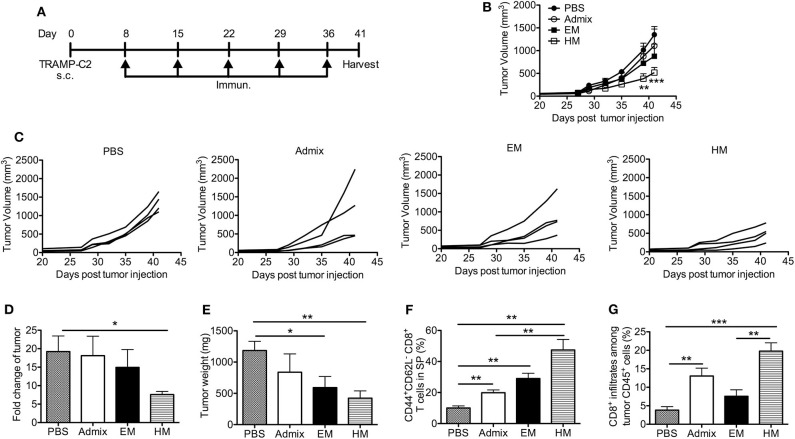
HM IS-SNAs inhibit TRAMP-C2 tumor growth. Mice (4 mice per group) inoculated subcutaneously with TRAMP-C2 tumor cells were immunized with admix, EM or HM IS-SNAs containing PAP and CpG every week for 5 times, tumors and spleens were harvested on day 41. **(A)** Treatment schedule for TRAMP-C2 tumor bearing mice. **(B)** Average and **(C)** individual tumor growth curve of TRAMP-C2 bearing mice. **p < 0.01 (day 39) and ***p < 0.001 (day 41) for 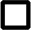 vs 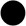; *p < 0.05 for 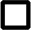 vs 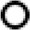 (day 41), calculated by two-way ANOVA with bonferroni multiple comparisons post-test. **(D)** The fold change of tumor was calculated using tumor volume of last measurement (day 41) divided by the 1st tumor measurement (day 27). **(E)** Tumor weight on day 41. **(F)** Percentage of CD44^+^CD62L^−^ CD8^+^ T cells in spleens on day 41. **(G)** Percentage of CD8^+^ T cells among CD45^+^ cells in tumor. All data are presented as mean ± SEM. *p < 0.05, **p < 0.01, and ***p < 0.001, analyzed by two-tailed unpaired Student's *t*-test **(D–G)**.

We evaluated the antitumor efficacy of SNAs formulated with CpG and PAP_115−123_ in the murine TRAMP-C2 prostate tumor model; TRAMP-C2 tumor cells express prostate acidic phosphatase (PAP) (39). Mice inoculated with TRAMP-C2 cells were immunized with HM and EM SNAs formulated with CpG and PAP_115−123_ every week for 5 weeks starting from day 8 (Figure 5A). Vaccination with the admix of CpG and PAP_115−123_ or EM SNAs did not show any significant inhibition of tumor growth compared with PBS control group (Figures 5B,C). In contrast, vaccination with HM SNAs led to significant tumor growth inhibition compared with the PBS group on Day 39 and 41 (*p* < 0.01, *p* < 0.001). We observed major differences between PBS and HM SNA groups in the fold change of tumor volume between first and last measurements (18-fold increase for the PBS group, 7-fold increase for HM SNA-vaccinated mice) (*p* < 0.05, Figure 5D), and the weight of the tumors at the end point of day 41 (Figure 5E). Mice vaccinated with EM SNAs also led to weights of tumors that were lower than those for PBS treated mice (*p* < 0.05). Analysis of the T cells in the tumor-bearing mice showed that the percentage of CD44^+^CD62L^−^ effector memory CD8^+^ T cells was greater for mice vaccinated with HM SNAs, when compared with admix treated group (*p* < 0.01, Figure 5F). The percentage of tumor infiltrating CD8^+^ T cells was higher in the HM SNA group than in PBS control group (Figure 5G). Taken together, the data show that HM SNAs are the most effective therapeutic vaccine among the groups tested against TRAMP-C2 tumors.

**Figure 6:**
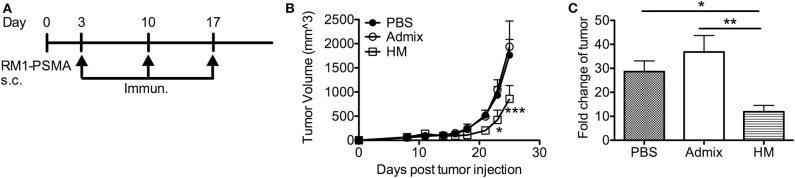
HM IS-SNAs delayed the tumor growth of RM1-PSMA. Mice (3–5 mice per group) inoculated subcutaneously with 10^5^ RM1-PSMA tumor cells were immunized with admix, HM IS-SNAs containing PSMA_634−642_ and CpG on day 3, 10, 17. **(A)** Treatment schedule for RM1-PSMA tumor bearing mice. **(B)** Tumor growth curve of RM1-PSMA bearing mice. ***p < 0.001 (day 25) for 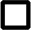 vs 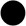; *p < 0.05 (day 23) and ***p < 0.001 (day 25) for 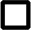 vs 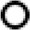, calculated by two-way ANOVA with bonferroni multiple comparisons post-test. **(C)** The fold change of tumor was calculated using tumor volume of last measurement divided by the 1st tumor measurement. *p < 0.05 and **p < 0.01, analyzed by two-tailed unpaired Student's *t*-test. All data are presented as mean ± SEM.

We further evaluated the anti-tumor efficacy of HM SNAs in a tumor model featuring PSMA (RM1-PSMA); PSMA is well-known as a TAA that is overexpressed in prostate cancer. RM1 is a highly aggressive, poorly immunogenic cancer cell line (40). Mice receiving subcutaneous injections of RM1-PSMA cells were immunized weekly starting from day 3 with HM SNAs formulated with CpG and PSMA_634−642_ or an admix formulation. Mice from the admix group showed rapid tumor growth and no benefit over the untreated group (Figures 6A,B). Vaccination with HM SNAs however led to delayed tumor growth. The tumor volume was significantly smaller on days 23 and 25 compared with that for the admix group (*p* < 0.05, *p* < 0.001, Figure 6B). The difference was also reflected by the fold change of tumor volume between first and last measurements (Figure 6C).

## Discussion

Prostate cancer tumors are weakly immunogenic (41), and the fact that immune checkpoint blockade therapy has failed as monotherapy for prostate cancer suggests the need for other approaches to immunotherapy (42). Therapeutic vaccines for prostate cancer (cell-based vaccines such as Sipuleucel-T, and viral/DNA-based vaccines such as PROSTVAC) have shown limited benefits clinically; the urgent need for innovation in vaccine immunotherapy for prostate cancer persists. In this study, we have shown that the adaptation of IS-SNA structures, by formulating them with MHC class I-restricted peptides derived from prostate tumor-associated antigens, leads to a novel therapeutic platform that can function as a cancer vaccine capable of generating antigen-specific T cell response against murine prostate cancer models. Our study here has now shown the ability of SNA structures, particularly HM SNAs, to raise immune responses against antigens (PSA, PSMA, PAP) that are clinically relevant for treating prostate cancer and is consistent with what has been observed with clinically irrelevant model antigens (25).

The selection of adjuvant is a key component in the design of a cancer vaccine; the adjuvant plays an important role in breaking immune tolerance, and directing the type of immune responses induced against antigens. CpG oligonucleotides drive strong Th1 type immune response (43) and the development of therapeutic vaccination strategies using CpG as an adjuvant continues, with recent preclinical studies showing promising results in multiple cancer types (8, 44). SNAs are structures that enhance the immunestimulatory activity of CpG, and the development of an SNA formulation of CpG has advanced to clinical trials (24). Our design of SNA vaccines for prostate cancer thus uses CpG as adjuvant and focuses on the type and placement of prostate cancer-associated antigens within SNA structures for raising antigen-specific immune responses. Our use of class B CpG in SNAs in this study (and others), takes advantage of the ability of class B CpG to induce DC maturation and production of Th1 cytokines, such as IL-12, which is critical in the process of CTL priming (35, 44).

Importantly, the modularity of SNAs has allowed us to formulate two classes of structures with nearly identical amounts of antigen and adjuvant but with varied presentations (EM and HM structures). The data underscore the importance of SNA structure on the quality of CTL responses. Specifically, HM SNAs (in which the peptide is chemically conjugated to oligonucleotides and associated to SNAs through nucleic acid hybridization) elicited remarkably stronger immune responses than EM SNAs (in which the peptide antigen is encapsulated within the liposomal core of SNAs). These outcomes are correlated with the enhanced co-delivery of antigen and CpG to DCs in dLNs (45). In HM SNAs, the association of peptide to SNAs by chemical conjugation led to higher levels of antigen uptake than that of EM SNAs; the design of HM SNAs and the chemical conjugation of peptide to oligonucleotides enables the biochemical release of antigen within DCs, a key requirement for cross-priming T cells. The improvement in the quality of anti-tumor immune responses observed in HM SNAs indicates the potential for further advances in SNA vaccine design, through rational changes in SNA structure informed by observations of cellular uptake and processing of CpG and antigen by APCs, and to ultimately translate this technology in cancer vaccine immunotherapy to prostate cancer patients.

While significant, the anti-tumor responses raised by HM SNAs did not eradicate the tumor in the selected prostate cancer models. This is likely a consequence of several factors: (1) the tumor models used here (particularly RM1) are insufficient in their expression of MHC class I molecules (46, 47), limiting antigen-specific T cell recognition and attack; (2) immunosuppressive mechanisms mounted by tumors including the induction or expansion of immunosuppressive cells and the up-regulation of T cell co-inhibitory molecules (e.g., PD-1/PD-L1), hinder the anti-tumor T cell response; (3) the time required to generate high numbers of antigen-specific T cells limits the ability of therapeutic vaccination to control rapidly growing tumors. This factor may not be significant in human disease, where the clinical observation of prostate cancer often finds slow growth and progression; (4) insufficient impact of targeting a single epitope. Loss of antigen occurs as tumors progress, comprising a mechanism to escape antigen-specific immune responses, one induced by selective pressure (48). Taken together, our observations in this study provide the basis for designing and optimizing SNAs in future approaches to augment the antitumor T cell immune responses through combination with immune checkpoint blockade therapy (e.g., anti-PD-1/PD-L1) and by raising broad-spectrum T cell immune responses to both MHC class I and MHC class II-restricted antigens simultaneously through multiplexed SNAs that can deliver multiple epitopes. Our study here has revealed the importance of structure and SNA design in therapeutic vaccination and improving the primary antigen-specific CTL responses relevant to prostate cancer tumors. While outside of the scope of our study here, long term protective effects are also important and desired from therapeutic vaccination. These effects are particularly relevant for protection against relapse and metastasis and should be examined in the study of SNA vaccines in ongoing and future development of therapeutic vaccination for prostate cancer.

In conclusion, IS-SNAs are scalable clinically, containing multivalent properties to manipulate, and induce strong antigen-specific immune responses. All these characters make it a promising candidate for clinical translation. Furthermore, development of the structural IS-SNAs conjugated with multiple tumor associate antigens and/or tumor neoantigens appears to be a powerful and versatile approach for treatment of different types of cancer, which is also crucial for personalized cancer immunotherapy.

## Data Availability Statement

The datasets generated for this study are available on request to the corresponding author.

## Ethics Statement

The animal study was reviewed and approved by Institutional Animal Use Committee at Northwestern University.

## Author Contributions

LQ, SW, and DD conducted the experiments. LQ, SW, ALe, CM, and BZ designed the experiments. LQ, ALe, and BZ wrote the paper. ALe, CM, and BZ edited the paper. ALo, SC, JF, JA, KS, and ZH provide materials and tools. LQ, SW, ZH, and BZ analyzed data. ALe, CM, and BZ supervised the study. All authors contributed to the article and approved the submitted version.

## Conflict of Interest

CM has financial interests in Exicure, Inc., who licenses SNA technology and could potentially benefit from the outcomes of this research. The remaining authors declare that the research was conducted in the absence of any commercial or financial relationships that could be construed as a potential conflict of interest.

## References

[1] Siegel RL Miller KD Jemal A Cancer statistics, 2020. CA Cancer J Clin. (2020) 70:7–30. 10.3322/caac.21590 31912902

[2] Attard G de Bono JS. Translating scientific advancement into clinical benefit for castration-resistant prostate cancer patients. Clin Cancer Res. (2011) 17:3867–75. 10.1158/1078-0432.CCR-11-0943 21680542

[3] Yu S Jia L Zhang Y Wu D Xu Z Ng CF. Increased expression of activated endothelial nitric oxide synthase contributes to antiandrogen resistance in prostate cancer cells by suppressing androgen receptor transactivation. Cancer Lett. (2013) 328:83–94. 10.1016/j.canlet.2012.09.006 22995070

[4] Schweizer MT Drake CG. Immunotherapy for prostate cancer: recent developments and future challenges. Cancer Metastasis Rev. (2014) 33:641–55. 10.1007/s10555-013-9479-8 24477411 PMC4116461

[5] Kantoff PW Higano CS Shore ND Berger ER Small EJ Penson DF. Sipuleucel-T immunotherapy for castration-resistant prostate cancer. N Engl J Med. (2010) 363:411–22. 10.1056/NEJMoa1001294 20818862

[6] Maeng H Terabe M Berzofsky JA. Cancer vaccines: translation from mice to human clinical trials. Curr Opin Immunol. (2018) 51:111–22. 10.1016/j.coi.2018.03.001 29554495 PMC5943163

[7] Gulley JL Borre M Vogelzang NJ Ng S Agarwal N Parker CC Phase III trial of PROSTVAC in asymptomatic or minimally symptomatic metastatic castration-resistant prostate cancer. J Clin Oncol. (2019) 37:1051–61. 10.1200/JCO.18.02031 30817251 PMC6494360

[8] Kuai R Ochyl LJ Bahjat KS Schwendeman A Moon JJ. Designer vaccine nanodiscs for personalized cancer immunotherapy. Nat Mater. (2017) 16:489–96. 10.1038/nmat4822 28024156 PMC5374005

[9] Melief CJ van der Burg SH. Immunotherapy of established (pre)malignant disease by synthetic long peptide vaccines. Nat Rev Cancer. (2008) 8:351–60. 10.1038/nrc2373 18418403

[10] Hailemichael Y Dai ZM Jaffarzad N Ye Y Medina MA Huang XF. Persistent antigen at vaccination sites induces tumor-specific CD8(+) T cell sequestration, dysfunction and deletion. Nat Med. (2013) 19:465–72. 10.1038/nm.3105 23455713 PMC3618499

[11] Zheng D Giljohann DA Chen DL Massich MD Wang XQ Iordanov H. Topical delivery of siRNA-based spherical nucleic acid nanoparticle conjugates for gene regulation. Proc Natl Acad Sci USA. (2012) 109:11975–80. 10.1073/pnas.1118425109 22773805 PMC3409786

[12] Jensen SA Day ES Ko CH Hurley LA Luciano JP Kouri FM. Spherical nucleic acid nanoparticle conjugates as an RNAi-based therapy for glioblastoma. Sci Transl Med. (2013) 5:209ra152. 10.1126/scitranslmed.3006839 24174328 PMC4017940

[13] Liu HM Kang RS Bagnowski K Yu JM Radecki S Daniel WL. Targeting the IL-17 receptor using liposomal spherical nucleic acids as topical therapy for psoriasis. J Invest Dermatol. (2020) 140:435–44. 10.1016/j.jid.2019.06.146 31421125 PMC8375607

[14] Radovic-Moreno AF Chernyak N Mader CC Nallagatla S Kang RS Hao L. Immunomodulatory spherical nucleic acids. Proc Natl Acad Sci USA. (2015) 112:3892–7. 10.1073/pnas.1502850112 25775582 PMC4386353

[15] Randeria PS Seeger MA Wang XQ Wilson H Shipp D Mirkin CA. siRNA-based spherical nucleic acids reverse impaired wound healing in diabetic mice by ganglioside GM3 synthase knockdown. Proc Natl Acad Sci USA. (2015) 112:5573–8. 10.1073/pnas.1505951112 25902507 PMC4426446

[16] Skakuj K Wang S Qin L Lee A Zhang B Mirkin CA. Conjugation chemistry-dependent T-cell activation with spherical nucleic acids. J Am Chem Soc. (2018) 140:1227–30. 10.1021/jacs.7b12579 29356509 PMC5831183

[17] Giljohann DA Seferos DS Patel PC Millstone JE Rosi NL Mirkin CA. Oligonucleotide loading determines cellular uptake of DNA-modified gold nanoparticles. Nano Lett. (2007) 7:3818–21. 10.1021/nl072471q 17997588 PMC8585332

[18] Patel PC Giljohann DA Daniel WL Zheng D Prigodich AE Mirkin CA. Scavenger receptors mediate cellular uptake of polyvalent oligonucleotide-functionalized gold nanoparticles. Bioconjug Chem. (2010) 21:2250–6. 10.1021/bc1002423 21070003 PMC3241523

[19] Cutler JI Auyeung E Mirkin CA. Spherical nucleic acids. J Am Chem Soc. (2012) 134:1376–91. 10.1021/ja209351u 22229439

[20] Choi CHJ Hao LL Narayan SP Auyeung E Mirkin CA. Mechanism for the endocytosis of spherical nucleic acid nanoparticle conjugates. Proc Natl Acad Sci USA. (2013) 110:7625–30. 10.1073/pnas.1305804110 23613589 PMC3651452

[21] Narayan SP Choi CHJ Hao LL Calabrese CM Auyeung E Zhang C. The sequence-specific cellular uptake of spherical nucleic acid nanoparticle conjugates. Small. (2015) 11:4173–82. 10.1002/smll.201500027 26097111 PMC4560454

[22] Parmiani G Castelli C Dalerba P Mortarini R Rivoltini L Marincola FM. Cancer immunotherapy with peptide-based vaccines: what have we achieved? Where are we going? J Natl Cancer Inst. (2002) 94:805–18. 10.1093/jnci/94.11.805 12048268

[23] Shirota H Tross D Klinman DM. CpG oligonucleotides as cancer vaccine adjuvants. Vaccines. (2015) 3:390–407. 10.3390/vaccines3020390 26343193 PMC4494345

[24] Exicure Inc. (2019). Intratumoral AST-008 Combined With Pembrolizumab in Patients with Advanced Solid Tumors. Available online at: https://clinicaltrials.gov/ct2/show/NCT03684785 (accessed June 17, 2020).

[25] Wang S Qin L Yamankurt G Skakuj K Huang Z Chen PC. Rational vaccinology with spherical nucleic acids. Proc Natl Acad Sci USA. (2019) 116:10473–81. 10.1073/pnas.1902805116 31068463 PMC6535021

[26] Banga RJ Chernyak N Narayan SP Nguyen ST Mirkin CA. Liposomal spherical nucleic acids. J Am Chem Soc. (2014) 136:9866–9. 10.1021/ja504845f 24983505 PMC4280063

[27] Medin JA Liang SB Hou JWS Kelley LS Peace DJ Fowler DH. Efficient transfer of PSA and PSMA cDNAs into DCs generates antibody and T cell antitumor responses *in vivo*. Cancer Gene Ther. (2005) 12:540–51. 10.1038/sj.cgt.7700810 15678150

[28] Williams BJ Bhatia S Adams LK Boling S Carroll JL Li XL. Dendritic cell based PSMA immunotherapy for prostate cancer using a CD40-targeted adenovirus vector. PLoS ONE. (2012) 7:e46981. 10.1371/journal.pone.0046981 23056548 PMC3466199

[29] Klyushnenkova EN Kouiavskaia DV Berard CA Alexander RB. Cutting edge: permissive MHC class II allele changes the pattern of antitumor immune response resulting in failure of tumor rejection. J Immunol. (2009) 182:1242–6. 10.4049/jimmunol.182.3.1242 19155468 PMC2743899

[30] Saif JM Vadakekolathu J Rane SS McDonald D Ahmad M Mathieu M. Novel prostate acid phosphatase-based peptide vaccination strategy induces antigen-specific T-cell responses and limits tumour growth in mice. Eur J Immunol. (2014) 44:994–1004. 10.1002/eji.201343863 24338683

[31] Qin L Dominguez D Chen S Fan J Long A Zhang M. Exogenous IL-33 overcomes T cell tolerance in murine acute myeloid leukemia. Oncotarget. (2016) 7:61069–80. 10.18632/oncotarget.11179 27517629 PMC5308636

[32] Zhang B Karrison T Rowley DA Schreiber H. IFN-γ- and TNF-dependent bystander eradication of antigen-loss variants in established mouse cancers. J Clin Invest. (2008) 118:1398–404. 10.1172/JCI33522 18317595 PMC2262029

[33] Oh S Hodge JW Ahlers JD Burke DS Schlom J Berzofsky JA. Selective induction of high avidity CTL by altering the balance of signals from APC. J Immunol. (2003) 170:2523–30. 10.4049/jimmunol.170.5.2523 12594278

[34] Su Z Wang X Zheng L Lyu T Figini M Wang B. MRI-guided interventional natural killer cell delivery for liver tumor treatment. Cancer Med. (2018) 7:1860–9. 10.1002/cam4.1459 29601672 PMC5943467

[35] Sabado RL Bhardwaj N. Directing dendritic cell immunotherapy towards successful cancer treatment. Immunotherapy. (2010) 2:37–56. 10.2217/imt.09.43 20473346 PMC2867472

[36] Moynihan KD Holden RL Mehta NK Wang C Karver MR Dinter J. Enhancement of peptide vaccine immunogenicity by increasing lymphatic drainage and boosting serum stability. Cancer Immunol Res. (2018) 6:1025–38. 10.1158/2326-6066.CIR-17-0607 29915023 PMC6247902

[37] Yewdall AW Drutman SB Jinwala F Bahjat KS Bhardwaj N. CD8+ T cell priming by dendritic cell vaccines requires antigen transfer to endogenous antigen presenting cells. PLoS ONE. (2010) 5:e11144. 10.1371/journal.pone.0011144 20585396 PMC2886840

[38] Henry CJ Ornelles DA Mitchell LM Brzoza-Lewis KL Hiltbold EM. IL-12 produced by dendritic cells augments CD8(+) T cell activation through the production of the chemokines CCL1 and CCL17. J Immunol. (2008) 181:8576–84. 10.4049/jimmunol.181.12.8576 19050277 PMC2716729

[39] Castelo-Branco P Passer BJ Buhrman JS Antoszczyk S Marinelli M Zaupa C. Oncolytic herpes simplex virus armed with xenogeneic homologue of prostatic acid phosphatase enhances antitumor efficacy in prostate cancer. Gene Ther. (2010) 17:805–10. 10.1038/gt.2010.20 20220784 PMC3243306

[40] Huang X Raskovalova T Lokshin A Krasinskas A Devlin J Watkins S. Combined antiangiogenic and immune therapy of prostate cancer. Angiogenesis. (2005) 8:13–23. 10.1007/s10456-005-2893-y 16132614

[41] Varghese S Rabkin SD Nielsen PG Wang W Martuza RL. Systemic oncolytic herpes virus therapy of poorly immunogenic prostate cancer metastatic to lung. Clin Cancer Res. (2006) 12:2919–27. 10.1158/1078-0432.CCR-05-1187 16675589

[42] Goswami S Aparicio A Subudhi SK. Immune checkpoint therapies in prostate cancer. Cancer J. (2016) 22:117–20. 10.1097/PPO.0000000000000176 27111907 PMC4847149

[43] Krieg AM. Development of TLR9 agonists for cancer therapy. J Clin Invest. (2007) 117:1184–94. 10.1172/JCI31414 17476348 PMC1857270

[44] de Titta A Ballester M Julier Z Nembrini C Jeanbart L van der Vlies AJ. Nanoparticle conjugation of CpG enhances adjuvancy for cellular immunity and memory recall at low dose. Proc Natl Acad Sci USA. (2013) 110:19902–7. 10.1073/pnas.1313152110 24248387 PMC3856841

[45] Nierkens S den Brok MH Sutmuller RP Grauer OM Bennink E Morgan ME. *In vivo* colocalization of antigen and CpG [corrected] within dendritic cells is associated with the efficacy of cancer immunotherapy. Cancer Res. (2008) 68:5390–6. 10.1158/0008-5472.CAN-07-6023 18593941

[46] Bander NH Yao D Liu H Chen YT Steiner M Zuccaro W. MHC class I and II expression in prostate carcinoma and modulation by interferon-alpha and -gamma. Prostate. (1997) 33:233–9. 10.1002/(SICI)1097-0045(19971201)33:4<233::AID-PROS2>3.0.CO 9397194

[47] Simons JW. Prostate cancer immunotherapy: beyond immunity to curability. Cancer Immunol Res. (2014) 2:1034–43. 10.1158/2326-6066.CIR-14-0174 25367978

[48] Vyas M Muller R Pogge von Strandmann E. Antigen loss variants: catching hold of escaping foes. Front Immunol. (2017) 8:175. 10.3389/fimmu.2017.00175 28286501 PMC5323381

