# Supplementary material for: Development of Spherical Nucleic Acids for Prostate Cancer Immunotherapy
Source: Front Immunol. 2020 Jul 8;11:1333. doi: 10.3389/fimmu.2020.01333 (PMC7362897; doi:10.3389/fimmu.2020.01333)
Supplement: Supplementary file 1 [file Data_Sheet_1.docx]

Supplementary Material

# Supplementary Figures


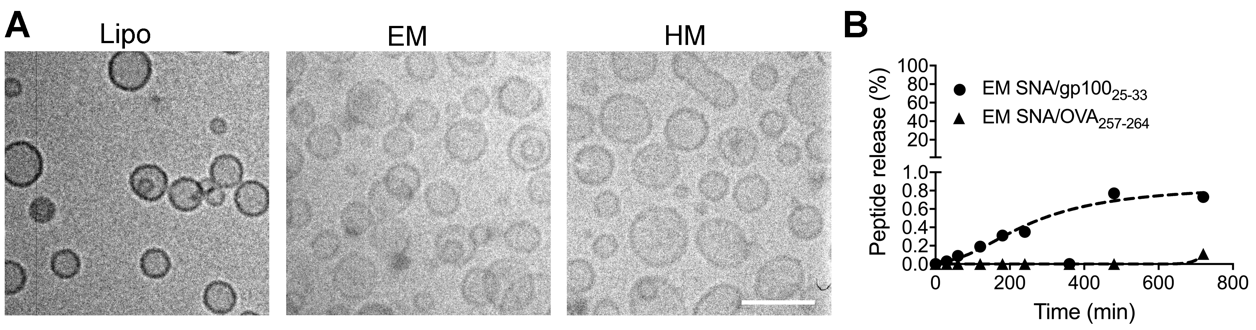


**Supplementary Figure 1**. Characterization of SNAs. (A) Cryo-electron microscopy images of liposomes, EM and HM IS-SNAs. Scale bar, 100 nm. (B) Leakage of peptide from EM SNAs. Two kinds of FITC labeled MHC class I restricted peptides were encapsulated into SNAs. And the leakage was tested in the presence of 10% FBS at 37°C by increase of the fluorescence intensity.


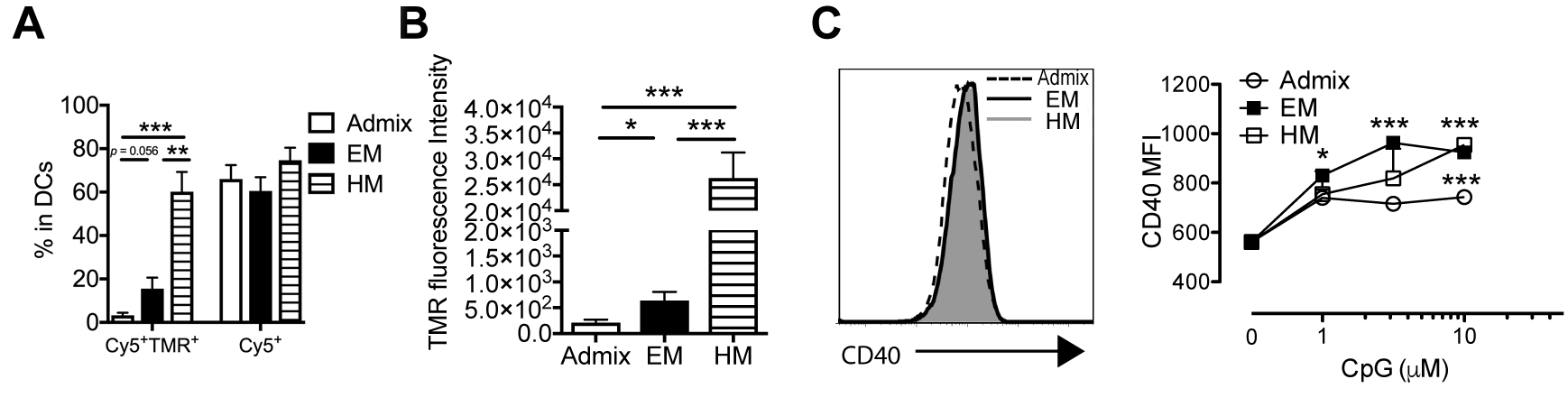


**Supplementary Figure 2**. IS-SNAs facilitate DC uptake and activation *in vitro*. (A) Percentages of Cy5^+^TMR^+^, Cy5^+^BMDCs after treated with IS-SNAs containing 25 nM CpG for 1 hour were analyzed by flow cytometry. (B) Mean fluorescence intensity (read out for confocal microscopy) of TMR-PSA_65-73_ taken up by BMDCs after culture for 1 h with SNAs. n= 7-9. (C) Median fluorescent intensity (MFI) and representative histograms for CD40 expression by BMDCs treated with IS-SNAs. DCs were incubated with IS-SNAs for 0.5 hour, and the CD40 expression level was quantified eight hours after initiating incubation. Mean ± SEM. **p* < 0.05, ****p* < 0.001 when comparing with admix, analyzed by two-tailed unpaired Student’s t-test.


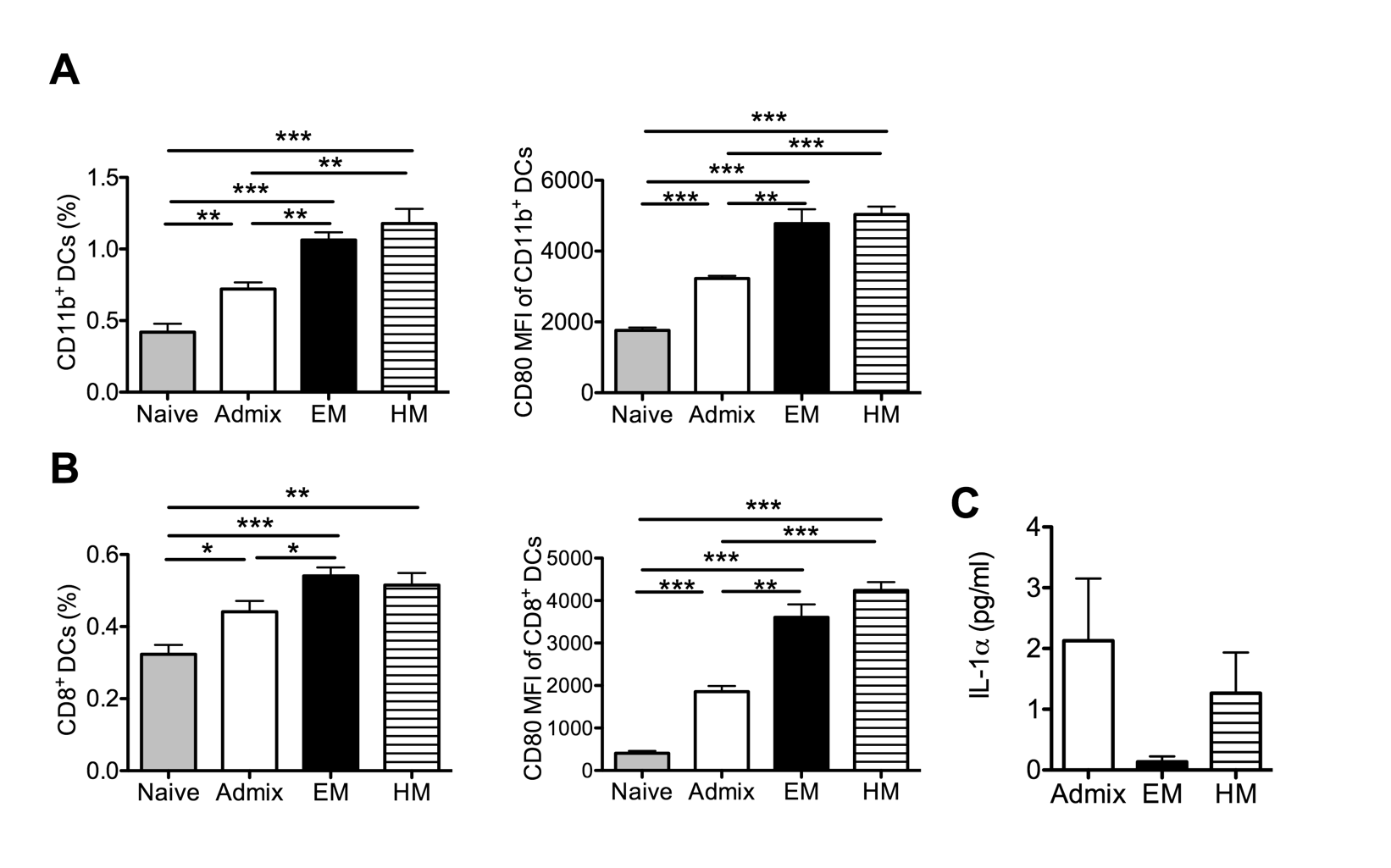


**Supplementary Figure 3**. IS-SNAs promote DC activation *in vivo*. Mice were administered with PSA_65-73_ and CpG in the form of admix, EM or HM IS-SNAs, dLNs were harvested 24 hours later and DCs were analyzed by flow cytometry. n=4. (A) Percentage of CD11b^+^ DCs and the expression of co-stimulatory molecule CD80 on CD11b^+^ DCs. (B) Percentage of CD8^+^ DCs and the expression of co-stimulatory molecule CD80 on CD8^+^ DCs. (C) The IL-1α production by DCs was analyzed from the supernatant of DC and T cell co-culture. All data are presented as mean ± SEM. *p < 0.05, **p < 0.01, ***p < 0.001, analyzed by two-tailed unpaired Student’s t-test.


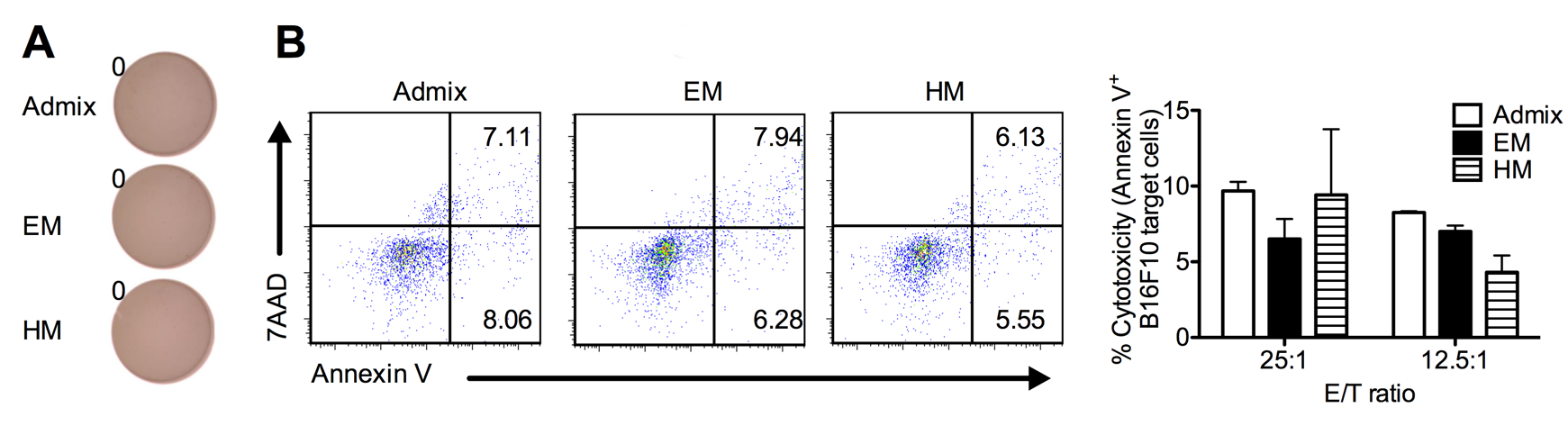


**Supplementary Figure 4.** IS-SNA promote PSA antigen specific CD8^+^ T cell response. (A) Splenocytes isolated from mice immunized 3 times with admix or IS-SNA/PSA_65-73_ were stimulated with irrelevant peptide gp100 for 48 hours. ELISPOT assay was used to measure IFN-γ secreting cells. (B) Splenic CD8^+^ T cell cytotoxicity to cells without PSA expression (B16F10). Left, representative flow dot plots at 12.5:1 E/T ratio; Right, summary of CD8^+^ T cell cytotoxicity reflected by the percentage of apoptotic target cells. All data are presented as mean ± SEM.


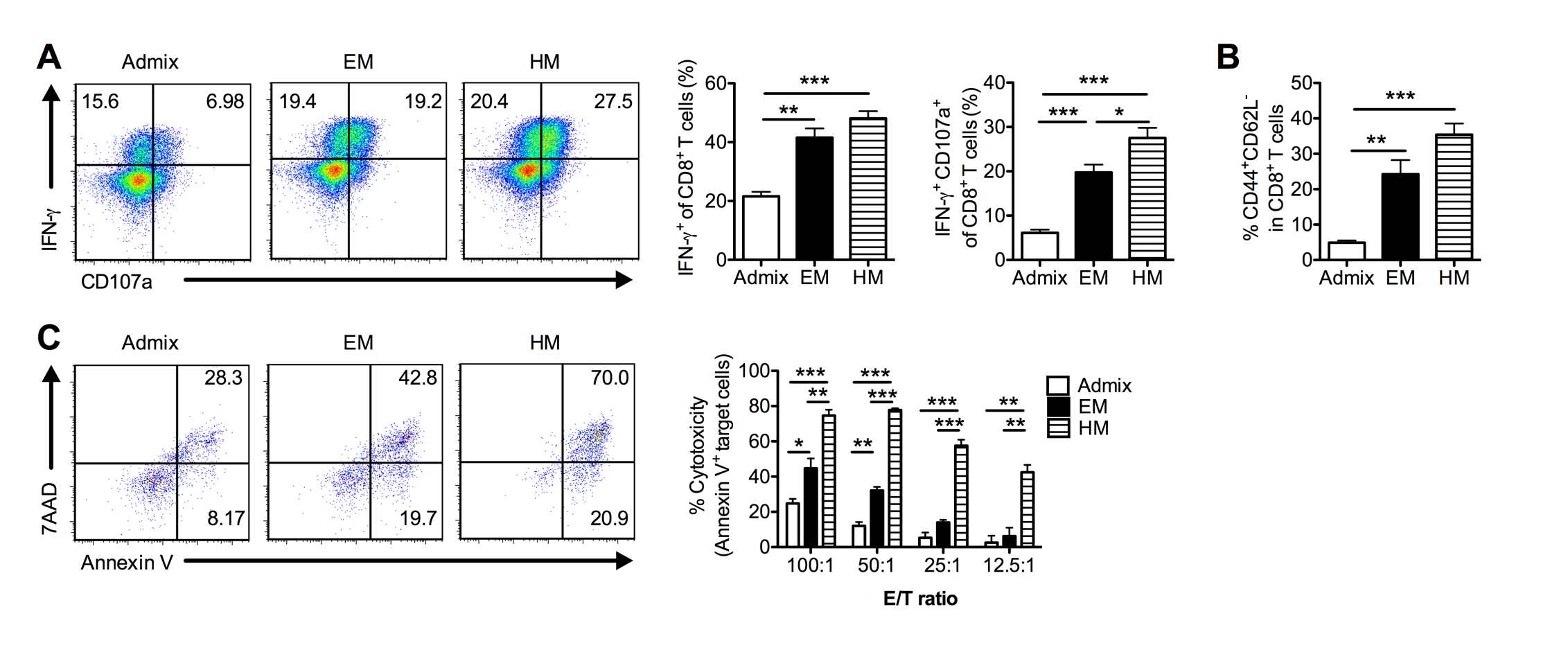


**Supplementary Figure 5.** IS-SNAs promote PSMA antigen specific CD8^+^ T cell responses. Mice were subcutaneously immunized with EM, HM IS-SNA/PSMA_634-642_ or admix of PSMA_634-642_ and CpG formulation every 2 weeks for 3 times. One week after final immunization spleens were harvested for analysis. n = 4. (A) Representative flow dot plots and bar graph of IFN-γ and CD107a expression in splenic CD8^+^ T cells. (B) Percentage of CD44^+^CD62L^-^ CD8^+^ T cells in spleens after immunization. (C) Splenic CD8^+^ T cell cytotoxicity to target cell RM1-PSMA at different effector/target (E/T) ratio. Left, representative flow dot plots at 100:1 E/T ratio; Right, summary of CD8^+^ T cell cytotoxicity reflected by the percentage of apoptotic target cells. All data are presented as mean ± SEM. **p* < 0.05, ***p* < 0.01 and ****p* < 0.001, analyzed by two-tailed unpaired Student’s t-test.


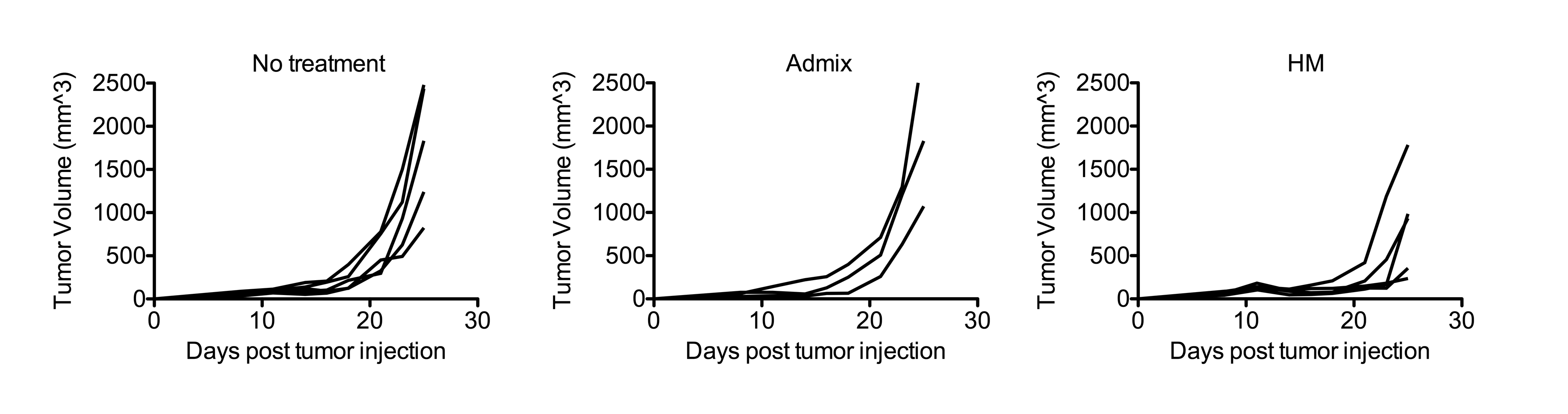


**Supplementary Figure 6.** Individual tumor growth curve of RM1-PSMA.
